# Burden of laboratory-confirmed shigellosis infections in Guatemala 2007-2012: results from a population-based surveillance system

**DOI:** 10.1186/s12889-019-6780-7

**Published:** 2019-05-10

**Authors:** Sonia Hegde, Stephen R. Benoit, Wences Arvelo, Kim Lindblade, Beatriz López, John P. McCracken, Chris Bernart, Aleida Roldan, Joe P. Bryan

**Affiliations:** 1 Centers for Disease Control and Prevention, Atlanta, GA USA; 2 Johns Hopkins University, Baltimore, MD USA; 3 Centro de Estudios en Salud, Universidad del Valle de Guatemala, Guatemala City, Guatemala

**Keywords:** *Shigella*, Guatemala, Epidemiology, Antimicrobial resistance, Global health security

## Abstract

**Background:**

We describe the epidemiology and antimicrobial susceptibility patterns of culture-confirmed *Shigella* infections in facility-based surveillance sites in Guatemala. Current studies using quantitative molecular diagnostics suggest *Shigella* may contribute most to the global diarrheal disease burden. Since identification of *Shigella* requires culturing techniques using stool specimens and few laboratories in Guatemala routinely culture for this pathogen, little is known about the true burden of *Shigella* in Guatemala or, importantly, the antimicrobial resistance patterns.

**Methods:**

Clinical, epidemiological, and laboratory data were collected on 5399 patients with acute diarrhea (≥3 loose stools in 24 h) from June 2007–August 2012. Multidrug resistance (MDR) was defined as resistance to ampicillin and trimethoprim/sulfamethoxazole.

**Results:**

Five percent (261) of stool specimens yielded *Shigella spp.* The annual incidence of laboratory-confirmed infections ranged from 5.0 to 24.1 per 100,000 persons in Santa Rosa and 0.3 to 6.2 per 100,000 in Quetzaltenango; 58% of cases occurred in children < 5 years of age. Thirty patients were hospitalized; one patient died. Oral rehydration or intravenous solution was used to treat 72% of hospitalized and 15% of ambulatory cases. Fifty-nine percent of cases were *S. flexneri* and 51% of cases were MDR.

**Conclusions:**

*Shigella* is an important cause of bacterial diarrhea in children and prevalence of MDR highlights the importance of appropriate treatment regimens. This study demonstrates that strengthening laboratory capacity in Guatemala can help determine causes which can lead to prevention of diarrheal diseases, particularly in children. Such capacity building is also critical for rapid detection and control of public health threats at their source and therefore for global health security.

**Electronic supplementary material:**

The online version of this article (10.1186/s12889-019-6780-7) contains supplementary material, which is available to authorized users.

## Background

Globally, diarrheal infections resulted in an estimated 688 million illnesses in 2015, and 499,000 deaths in children under 5 years of age [1, 2]. Despite dramatic reductions in childhood mortality in the past decade, diarrhea remains a major cause of preventable childhood deaths worldwide [3, 4], and still leads to higher risk of growth faltering and other health effects in those children that survive [5]. *Shigella* is one of four primary pathogens that substantially contributes to moderate and severe diarrhea globally [6, 7]. Indeed, quantitative molecular diagnostics suggest *Shigella* may contribute most to the global diarrheal disease burden [8]. While case fatalities have significantly decreased over time, morbidity from shigelloses remains an important problem and warrants investments in surveillance to better understand antibiotic resistance, treatment options, and preventive measures, including vaccine development [9].

Characterized by fever, abdominal pain, tenesmus, and loose stools, *Shigella* is usually acquired by ingestion of contaminated food or water [10]. In addition to the high mortality [11], sequelae of shigellosis can include encephalopathy, hemolytic uremic syndrome, bacteremia, dysentery, rectal prolapse [12], hypoglycemia, hyponatremia [13], and reactive arthritis [10, 14]. Children may experience high fever with convulsions [12]. Though previous studies have demonstrated the effectiveness of certain antimicrobials as treatment, resistance to commonly used antimicrobials such as trimethoprim/sulfamethoxazole, ampicillin, third generation cephalosporins, quinolones and macrolides [15–18] complicates treatment for those with severe disease [12, 19]. Additionally, fluoroquinolones, which may be effective, are not recommended for children and pregnant women [19]. Shigellosis may also be confused with acute amoebic dysentery, leading to inappropriate treatment with metronidazole or tinidazole [19]. Lastly, the diversity of *Shigella* serotypes has historically rendered vaccine development difficult [15].

In Guatemala, diarrhea remains the second most common cause of morbidity and mortality in children < 5 years of age [20], and small, community-based studies implicate *Shigella* as a common cause of diarrhea [21, 22]. However, since identification of *Shigella* requires culturing techniques using stool specimens [10] and few laboratories in Guatemala routinely culture for this pathogen, little is known about the true burden of *Shigella* in Guatemala or the antimicrobial resistance patterns. Limited laboratory capacity in Guatemala also results in delays in detecting, or an inability to recognize, outbreaks of *Shigella* infections*,* in addition to illnesses caused by other pathogens. Thus, controlling such outbreaks at their source is difficult, thereby threatening global health security. In this report, we characterize cases of laboratory-confirmed shigellosis enrolled in a healthcare facility-based surveillance system in Guatemala from 2007 through 2012 in order to inform prevention and treatment guidelines in Guatemala and the Central America region.

## Methods

### Study sites

In July 2007, the U.S. Centers for Disease Control and Prevention International Emerging Infections Program (CDC IEIP), the Guatemalan Ministry of Public Health and Welfare (MSPAS), and the Center for Health Studies of the Universidad del Valle de Guatemala (UVG) initiated an active facility-based surveillance system for diarrheal, respiratory, acute neurologic infections, and non-differentiated febrile illnesses in the department of Santa Rosa [23]. The surveillance system, Vigilancia Integrada Comunitaria (VICo), captured patients in both ambulatory and hospital settings. An additional site was added in February 2009 in the department of Quetzaltenango. Santa Rosa is located in the southern part of the country with a population of 346,590 in 2002, and Quetzaltenango is in the western highlands with a population of 789,358 [24]. Compared with Quetzaltenango, Santa Rosa has a lower elevation (1219 m vs. 2438 m at the hospitals), higher average temperature, higher average rainfall, and a smaller proportion of the population is Amerindian indigenous (15% versus 62%).

In Santa Rosa, the facility-based surveillance system included the Cuilapa Regional Hospital, one health center staffed by a physician and nurses, and five health posts, each staffed by a nurse. The Cuilapa Regional Hospital is a 176-bed referral hospital that provides care for residents of Santa Rosa and the neighboring departments of Jutiapa and Jalapa. In Quetzaltenango, the surveillance system included the Western Regional Hospital, three health centers, and one health post. The Western Regional Hospital is a 425-bed referral hospital that provides care for Quetzaltenango and the neighboring departments of Huehuetenango, San Marcos, Totonicapan, among other departments in Guatemala and Mexico. Health centers and health posts in both departments were considered ambulatory care. Private clinics, small private hospitals, pharmacies, traditional healers, and one public hospital in coastal Quetzaltenango make up the other sources of healthcare in the two departments.

### Case detection and data collection

A case of diarrhea was defined as ≥3 loose or liquid stools in a 24-h period with onset of illness within seven days before presenting to any participating facility. Inclusion criteria included residence in one of the municipalities included in the surveillance system. Cases with other diarrheal episodes within one week prior to the start of the current episode were excluded to avoid capturing illnesses due to chronic diarrhea. Surveillance nurses screened patients by reviewing logbook entries and assessed chief complaints for diarrhea-related visits and admissions. Detailed clinical, epidemiologic, demographic, and socioeconomic data were obtained from structured patient interviews and medical chart abstractions [25].

### Laboratory methods

All consenting patients were requested to provide bulk stool specimen. Rectal swabs were performed on children < 5 years of age who were unable to provide a specimen. Stool samples from the ambulatory facilities were then stored in Cary-Blair transport media at 4 °C and transported at 4 °C within 48 h to one of the two regional hospitals for initial processing and testing. Samples were subsequently streaked by direct plating onto selective agars, MacConkey and Xylose-lysine-desoxycholate (XLD) [26]. After incubation, colonies suggestive of *Shigella* (lactose negative colonies) were tested biochemically with Triple Sugar Iron (TSI), lysine iron (LIA), Sulfide-indole-motility (SIM) medium, Motility-indole-ornithine (MIO) medium, citrate, and urea. If the result of the biochemical test then suggested the colony examined was *Shigella* positive, the isolate was then tested with antisera for the different *Shigella* species.

At the hospital laboratories, antimicrobial susceptibility testing was performed on *Shigella* isolates using the disk diffusion method with the following antibiotics: ampicillin, amoxicillin, ceftriaxone, gentamicin, kanamycin, streptomycin, ciprofloxacin, nalidixic acid, chloramphenicol, trimethoprim/sulfamethoxazole and tetracycline, using Clinical Laboratory Standards Institute (CLSI) breakpoints [27]. Multidrug-resistance (MDR) was defined as resistance to at least ampicillin and trimethoprim/sulfamethoxazole, an important MDR phenotype for *Shigella* [11]. Samples were later sent to serotyping. The isolates were serotyped by slide agglutination tests using specific antisera at UVG laboratories (Denka Seiken Co, Ltd.).

### Data analysis

We examined the number of laboratory-confirmed *Shigella* cases identified in the surveillance system from July 2007 to August 2012 in Santa Rosa and from February 2009 to August 2012 in Quetzaltenango. We described the temperature and rainfall in each department during this period. Population-based annual crude incidence rates for *Shigella* were calculated by using the detected number of cases and catchment area population of the surveillance health facilities. Incidence rates were calculated from 2008 to 2012 for Santa Rosa, due to surveillance starting mid-year in 2007 in Santa Rosa. Incidence rates were calculated from 2009 to 2012 for Quetzaltenango. Population by municipality and age were obtained from the Guatemala National Institute of Statistics [24]. Population estimates for 2011 and 2012 were extrapolated from 2010 population data. As the catchment area for each surveillance site did not include all municipalities within a department, we used population estimates for only municipalities in our catchment area for the incidence denominator. The catchment area for the Cuilapa Regional Hospital included 11 municipalities in Santa Rosa Department, excluding the three coastal municipalities. The catchment area in Quetzaltenango included 10 municipalities, including Quetzaltenango city. Crude incidence rates were calculated across all ages and for children < 5 years of age. Poisson-based 95% confidence intervals were calculated for the annual rates of *Shigella*. The last year of surveillance was adjusted to account for eight months of observation. We also calculated adjusted incidence rates to account for differences in healthcare seeking behavior between hospitals and ambulatory care; we calculated different estimates of incidence based on cases that were detected at a hospital versus health center or post.

We described the demographic characteristics, symptoms, co-infections, and treatment of *Shigella* cases stratified by ambulatory and hospital settings for both Santa Rosa and Quetzaltenango. Chi-square or Fisher’s exact test were used to determine differences in characteristics between ambulatory and hospital settings. T-tests or analysis of variance (ANOVA) was used to test differences in means for continuous variables. We examined the number of days hospitalized patients spent in the intensive care unit, total length of hospital stay, and case fatality.

We characterized *Shigella* cases by serotype. Within each serotype, we counted the number of cases that were hospitalized and that reported bloody stool. Chi-square tests of proportions were used to determine differences across serotypes. Without comparative data from the underlying population at risk, we only assessed the proportion of *Shigella* cases according to potential risk factors, including education, safe water use, breastfeeding, refrigerator use, and large household size. Lastly, we reported the percent resistant to the antimicrobial classes and those with MDR. We also evaluated differences in antibiotic resistance between the surveillance sites and across time.

## Results

**Fig. 1:**
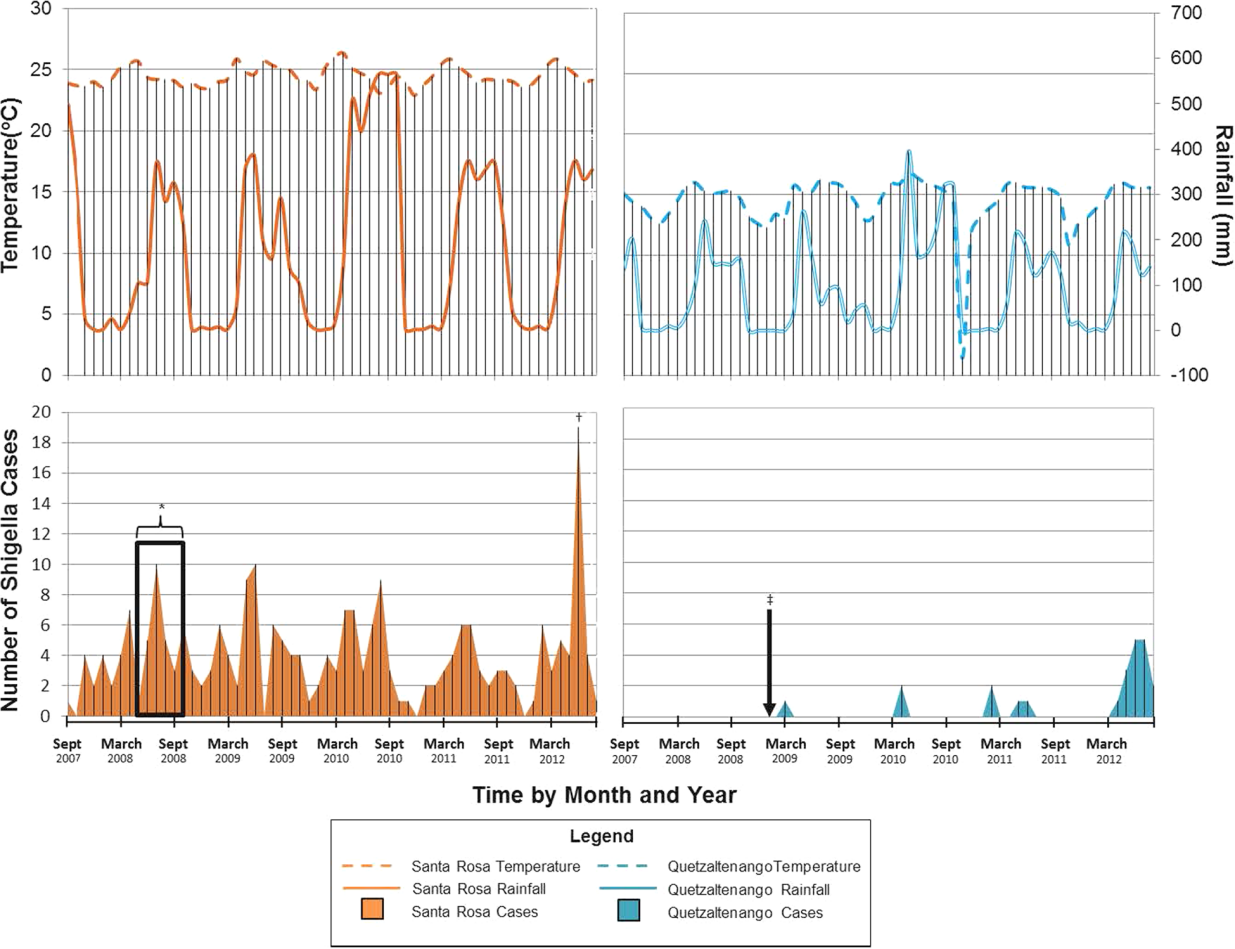
Seasonality of *Shigella* cases relative to average temperatures and monthly rainfall, 2007–2012 in Santa Rosa and Quetzaltenango Departments, Guatemala. *Peak season of cases and rainfall is from May to October. †This peak is largely from an outbreak at a single birthday party. ‡Surveillance started in February 2009 in Quetzaltenango.

From 2007 to 2012, stool samples from 5399 patients yielded 261 cases of Shigellosis, of which 91% were reported from the Santa Rosa department. Sixty-two percent of *Shigella* infections occurred from May to October (Fig. 1). Greater than half of all *Shigella* cases (58%) occurred in children < 5 years of age with a median age of 3 years (range: 1 month to 91 years); 149 (57%) were female (Table 1). Thirty (12%) patients with *Shigellosis* were hospitalized; 25 (83%) of the hospitalized patients were <  5 years of age and 4 (13%) were aged ≥60 years. Among ambulatory cases, 126 (55%) were <  5 years of age and 7 (3%) were aged ≥60 years. Hospitalized patients were significantly younger than ambulatory patients (*p* < 0.001). A greater proportion of hospitalized patients reported vomiting (*p* < 0.0001) and had convulsions and lethargy (*p* < 0.05). Parasites were observed in stool samples of 30% of patients with *Shigella*. Eighteen (72%) hospitalized cases were treated with oral rehydration salts or intravenous solution. Among hospitalized cases, the median hospital stay was 4 days. Six cases (20%) were admitted to the intensive care unit and one case died. The case that died was a one-year old child in Santa Rosa with *S. flexneri*; no co-morbidities or treatment regimen was reported for this case.

**Table 1:** Descriptive statistics of *Shigella* cases stratified by healthcare facility in Santa Rosa and Quetzaltenango Departments, Guatemala 2007–2012

	All Cases N (%)^a^ 261 (100)	Hospital N (%)^a^ 30 (100)	Ambulatory N (%)^a^ 231 (100)	*P-value*
Demographics				
*Sex*				
Female	149 (57.1)	14 (46.7)	135 (58.4)	0.22
*Age*				
0 to 6 months	2 (0.8)	1 (3.3)	1 (0.4)	0.001
6 months to < 1 year	16 (6.1)	5 (16.7)	11 (4.8)	
1 to 4 years	133 (50.9)	19 (63.2)	114 (49.5)	
5 to 9 years	36 (13.8)	1 (3.3)	35 (15.2)	
10 to 20 years	29 (11.1)	0 (0.0)	29 (12.6)	
20 to 40 years	25 (9.6)	0 (0.0)	25 (10.8)	
40 to 60 years	9 (3.5)	0 (0.0)	9 (3.9)	
60 + years	11 (4.2)	4 (13.3)	7 (3.0)	
Symptoms				
*Under 5 years*	*151 (100.0)*	*23 (100.0)*	*128 (100.0)*	
Vomiting	42 (28.0)	17 (74.0)	25 (37.8)	< 0.0001
Unable to drink or breastfeed	71 (47.0)	15 (60.0)	56 (44.1)	0.15
Convulsions	5 (4.0)	3 (13.0)	2 (2.2)	0.024
Lethargy	57 (36.0)	3 (12.0)	54 (40.0)	0.007
*All ages*				
Temperature in ^o^C *Median (Range)*	37 (34.4–40)	37 (36–39.5)	36.9 (34.4–40)	
Vomiting	99 (38.1)	21 (70.0)	78 (33.9)	< 0.0001
Abdominal Pain	185 (71.2)	11 (37.0)	174 (75.7)	< 0.0001
Bloody stool	80 (30.8)	5 (17.0)	75 (32.6)	0.19
Mucoid stool	183 (70.4)	15 (50.0)	168 (73.0)	0.009
Co-Infections				
Campylobacter coli	2 (0.8)	0 (0.0)	2 (0.9)	0.61
Campylobacter jejuni	4 (1.5)	0 (0.0)	4 (1.7)	0.47
Rotavirus	3 (1.3)	3 (10.0)	0 (0.0)	< 0.0001
Any protozoa or helminth	78 (30.0)	6 (20.0)	72 (31.6)	0.19
Giardia	5 (1.9)	0 (0.0)	5 (2.2)	0.41
Treatment^b^				
Oral rehydration salts or solution	37 (25.0)	18 (72.0)	19 (15.0)	< 0.0001

^a^Proportions based on non-missing data

^b^Not sufficient antibiotic data for treatment. 18 patients had other treatment, 4 had antibiotics and 9 had salts/solution

The education level of the parents of children < 5 years of age was low with 94% having no or only basic education (up to 9th grade). Overall, 67% of families reported monthly income of under $130/month. Only 37% of all cases reported having a refrigerator at home and over 60% of cases came from households of more than five people. In both the hospital and ambulatory setting, 78% of cases < 2 years were breastfed. A majority of cases reported using the public and municipal water sources, and also home storage of water.

The crude annual incidence rate of *Shigella* cases ranged from 13.8 to 24.1 per 100,000 from 2008 to 2012 in the Santa Rosa Department for all ages, and 52.3 to 91.9 per 100,000 persons for children < 5 years for both hospital and ambulatory cases (Table 2). Ambulatory care incidence estimates are higher for both children < 5 years and all ages than hospital care in Santa Rosa; the annual incidence rate of *Shigella* cases ranged from 348.2 to 594.5 cases for children < 5 years and 108.1 to 174.0 cases for all ages (Additional file 1). In Quetzaltenango, the crude annual incidence rates ranged from 0.3 to 6.2 per 100,000 persons from 2009 to 2012 for all ages, and 0 to 31.1 per 100,000 persons for children < 5 years (Table 2). The adjusted annual incidence in ambulatory care was as high as 92.5 cases for children < 5 years and 22.1 cases for all ages in Quetzaltenango (Additional file 1). In Quetzaltenango, there are more cases in 2012 than in previous years.

**Table 2:** Crude incidence rates of *Shigella* by site and age group in Santa Rosa and Quetzaltenango Departments, Guatemala, 2008–2012

	*Santa Rosa*		*Quetzaltenango* ^*b*^	
	Number of Cases	Crude Incidence Rate per 100,000 Population (95% CI)^a^	Number of Cases	Crude Incidence Rate per 100,000 Population (95% CI)^a^
*Overall*	231	15.1 (13.2, 17.1)	23	1.2 (0.76, 1.79)
*2008*				
*< 5 years*	34	91.9 (63.7, 128.4)		
*All ages*	52	21.1 (15.8, 27.7)		
*2009*				
*< 5 years*	32	85.6 (58.5, 120.8)	0	0.0 (0.0, 7.5)
*All ages*	54	21.5 (16.2, 28.1)	1	0.3 (0.008, 1.7)
*2010*				
*< 5 years*	26	68.8 (45.0, 100.8)	1	1.8 (0.05, 10.0)
*All ages*	46	18.0 (13.2, 24.0)	2	0.5 (0.07, 2.0)
*2011*				
*< 5 years*	20	52.3 (32.0, 80.8)	3	5.3 (1.1, 15.5)
*All ages*	36	13.8 (9.7, 19.1)	4	1.1 (0.29, 2.7)
*2012* ^*c*^				
*< 5 years*	18	69.7 (41.3, 110.1)	12	31.1 (16.1, 54.4)
*All ages*	43	24.1 (17.5, 32.5)	16	6.2 (3.5, 10.0)

^a^95% CI represents Upper Limit (UL), Lower Limit (LL)

^b^Surveillance in Quetzaltenango began in 2009

^c^Rates account for partial year through August 31

The most frequently isolated serotypes were *Shigella flexneri* (59%) and *Shigella sonnei* (36%). Among 30 hospitalized patients, 25 (83%) were infected with *S. flexneri* compared to 4 with *S. sonnei* (13%) and one with *S. boydii*. Among those cases with *S. flexneri*, 36% reported having bloody stool; 20% of *S. sonnei* cases reported having bloody stool.

Antimicrobial susceptibility testing was performed on 254 of the 261 laboratory-confirmed *Shigella* cases (Table 3). Resistance to ampicillin was detected in 61% of the isolates and resistance to trimethoprim/sulfamethoxazole in 83%; 120 (51%) cases were resistant to both (MDR). Sixty-three percent of cases in Santa Rosa were resistant to ampicillin as compared to 35% in Quetzaltenango, and 86% of cases in Santa Rosa were resistant to trimethoprim/sulfamethoxazole as compared to 52% in Quetzaltenango (Additional file 2). Fifty-five percent of cases were resistant to both ampicillin and trimethoprim/sulfamethoxazole (SMX/TMP) in Santa Rosa versus 13% in Quetzaltenango. Both resistance to ampicillin and MDR increased over the surveillance time period from 2007 to 2012 (Additional file 3). All isolates were resistant to at least one antimicrobial class tested; none were resistant to ciprofloxacin. Eighty percent of all isolates were resistant to at least three antimicrobial classes. There were no remarkable differences in antimicrobial resistance between hospital and ambulatory cases.

**Table 3:** Antimicrobial resistance by *Shigella* serotypes in Guatemala, 2007–2012

Antimicrobial class	Antimicrobial	All Cases N (%)^a^ *N* = 254	*S. soneii* N (%)^a^ *N* = 88	*S. flexneri* N (%)^a^ *N* = 152	*S. boydii* N (%)^a^ *N* = 12	*S. dysenteriae* N (%)^a^ *N* = 2
Aminoglycoside	*Gentamicin*	3 (1.0)	1 (1.0)	1 (1.0)	1 (8.0)	0 (0.0)
	*Kanamycin*	4 (2.0)	2 (3.0)	2 (2.0)	0 (0.0)	0 (0.0)
	*Streptomycin*	212 (85.0)	87 (99.0)	112 (76.0)	11 (92.0)	2 (100.0)
Beta-lactams	*Ampicillin*	141 (61.0)	45 (58.0)	87 (61.0)	8 (67.0)	1 (50.0)
	*Amoxicillin*	117 (47.0)	36 (41.0)	72 (49.0)	8 (67.0)	1 (50.0)
	*Ceftriaxone*	3 (2.0)	1 (2.0)	1 (1.0)	0 (0.0)	1 (50.0)
Quinolones	*Ciprofloxacin*	0 (0.0)	0 (0.0)	0 (0.0)	0 (0.0)	0 (0.0)
	*Nalidixic acid*	1 (0.4)	0 (0.0)	1 (1.0)	0 (0.0)	0 (0.0)
Phenicol	*Chloramphenicol*	37 (15.0)	2 (2.0)	35 (23.0)	0 (0.0)	0 (0.0)
Sulfonamide	*Trimethoprim sulfamethoxazole*	210 (83.0)	82 (93.0)	118 (78.0)	9 (75.0)	1 (50.0)
Tetracycline	*Tetracycline*	238 (96.0)	83 (94.0)	143 (97.0)	10 (91.0)	2 (100.0)
Level of resistance		All Cases N (%)^a^ *N* = 254	*S. soneii* N (%)^a^ *N* = 88	*S. flexneri* N (%)^a^ *N* = 152	*S. boydii* N (%)^a^ *N* = 12	*S. dysenteriae* N (%)^a^ *N* = 2
No resistance		0 (0.0)	0 (0.0)	0 (0.0)	0 (0.0)	0 (0.0)
Resistance to one antimicrobial class		15 (5.9)	3 (3.4)	12 (7.9)	0 (0.0)	0 (0.0)
Resistance to two antimicrobial classes		37 (14.6)	2 (2.3)	31 (20.4)	3 (25.0)	1 (50.0)
Resistance to three antimicrobial classes		66 (26.0)	39 (44.3)	24 (15.8)	3 (25.0)	0 (0.0)
Resistance to four antimicrobial classes		121 (47.6)	42 (47.7)	73 (48.0)	6 (50.0)	0 (0.0)
Resistance to five antimicrobial classes		15 (5.9)	2 (2.3)	12 (7.9)	0 (0.0)	1 (50.0)
Multidrug-resistance^b^		120 (51.3)	44 (56.4)	68 (47.9)	7 (58.3)	1 (50.0)

^a^Proportions based on non-missing data

^b^Resistance to ampicillin and trimethoprim-sulfamethoxazole (AT/S)

## Discussion

We characterized laboratory-confirmed *Shigella* cases captured by a facility-based surveillance system in Santa Rosa and Quetzaltenango, Guatemala. There are five characteristics among our findings that are pertinent to prevention and treatment of Shigellosis in Guatemala: age of infection, seasonality and region, species of *Shigella*, antimicrobial resistance, and antibiotic and rehydration treatments.

Most patients with *Shigella* presented to ambulatory care centers for treatment, and consistent with the known epidemiology of *Shigella,* we found the greatest burden of disease among young children [15]. Compared to the United States, the annual incidence of *Shigella* was higher for all ages and children < 5 years of age in Santa Rosa but not in Quetzaltenango [28, 29]. Incidence rates from active surveillance in Santa Rosa were 23.4–91.1 cases per 100,000 in children aged < 5 years compared with 16.9 cases per 100,000 reported in the U.S. [29]. Though higher than those of the U.S., rates in Guatemala were lower than those of Africa and Asia [30]. Still, the adjusted incidence estimates in this study revealed children < 5 years as an important age group, particularly in the ambulatory setting. Though children under 5 years comprised 58% of all cases and 80% of those hospitalized, 13% of those hospitalized were ≥ 60 years of age. Prevention of this food and waterborne infection must be targeted to protect the most vulnerable populations, that is, children and the elderly. Prevention measures like breastfeeding should be encouraged for children < 2 years, as recommended by the World Health Organization (WHO) [31].

There were large differences in the crude annual incidence rates of *Shigella* and antibiotic resistance patterns by department. Since higher temperatures allow bacteria to reproduce at a higher rate, the warm climate of Santa Rosa department may have contributed to the higher rate of food and waterborne disease [32, 33]. Moreover, most cases occurred during the warm, rainy season from May–October, indicating that special care must be taken in high-risk areas during this time to prevent transmission. Studies from China and Israel and the United States, as reported by the *National Antimicrobial Resistance Monitoring System for Enteric Bacteria* (NARMS) or other CDC surveillance, also found a seasonally higher rate of *Shigella* during periods of higher temperatures [33–35]. Such prevention tactics may include safe water and hygiene practices at the individual and household level, which for decades has been proven as an effective preventive measure for diarrheal disease [36, 37]. Special care in human feces disposal and prevention of transmission by hands, fomites and insects is essential. Low levels of education, however, and crowding at the household level may be obstacles to implementing prevention measures as these factors have been shown to increase diarrheal disease risk [38, 39]. Of note, the rates in Santa Rosa increased in 2012 as a result of an outbreak at a children’s birthday party involving 17 persons.

Similar to the rest of the world, the observed percentage of *S. flexneri* was approximately 60%; *S. flexneri* predominates in low-income countries [15]. However, *S. sonnei* (35%) is twice as high in our study as compared to global estimates for low-income countries (15%) [15]. Interestingly, in industrialized countries, *S. sonnei* is closer to 77% of *Shigella* infections while *S. flexneri* is 16% of *Shigella* infections, suggesting that Guatemala is in a developmental transition [15]. Fortunately, the epidemic of *Shigella dysenteriae* that affected much of Central America in the 1960s [40] and Guatemala in the 1990’s has abated [41]. Only one isolate of *S. dysenteriae* was detected in our surveillance.

Among isolates tested in our surveillance system in Guatemala, antimicrobial resistance to commonly used antimicrobials was high, as was the proportion that was resistant to at least three antimicrobial classes. The proportion of isolates that were resistant to at least ampicillin and SMX/TMP (51%) in Guatemala is markedly higher than in the United States (16%), as reported by NARMS in 2012 [11]. Because a majority of isolates of *Shigella* were resistant to accessible and inexpensive oral treatments like trimethoprim/sulfamethoxazole (83% resistant) and ampicillin (61% resistant), antimicrobial treatment options are limited in Guatemala. Though quinolones are effective and no resistance to ciprofloxacin was found in our study, quinolones have side effects such as joint and tendon injury, and are not generally used in children. In Bangladesh, there have been marked increases in resistance to ciprofloxacin [42]. This trend has also been noted in China, Canada, and the United States [11, 30, 43, 44]. Of note, resistance of *Shigella* to commonly used antibiotics in Guatemala, while greater than reported in the U.S. [11], is similar to that reported in other American countries including Mexico and Chile [43, 45].

While azithromycin is a first-line antibiotic treatment in the U.S. [16] and could be considered for treatment in Guatemala, it is important to note that resistance of *Shigella* to ampicillin and amoxicillin, both penem beta-lactam antibiotics, increased over the course of the surveillance period from 2007 to 2012. MDR resistance, resistance to both ampicillin and SMX/TMP, also increased over the surveillance period, limiting their therapeutic utility, especially in Santa Rosa. Continued surveillance of these dynamics is critical for understanding both prevention and treatment.

In addition to treatment with effective antibiotics, rehydration with oral rehydration solutions is a critical form of treatment [46]. Our findings, however, suggest that a substantial number of patients with Shigellosis were not treated with oral rehydration solution. Oral rehydration is an inexpensive and effective method that should be emphasized through education and policy.

Our findings are subject to limitations. These data apply only to the Departments of Santa Rosa and Quetzaltenango and therefore are not generalizable to all of Guatemala. However, they are the most comprehensive description of *Shigella* in Guatemala and Central America in the healthcare setting. As treatments are self-reported, information bias is a limitation of the reported use of rehydration therapies. Such bias, however, would indicate there are systematic differences among all cases and would neither over- or under-estimate treatment use. Furthermore, *Shigella* testing was done by standard cultures and not quantitative real-time polymerase chain reaction (PCR) due to resource constraints, suggesting the burden of disease estimates in this analysis are likely an underestimate of the true value [9]. By re-analyzing samples or subscribing to a quantitative real-time PCR detection method, the detection capability of *Shigella* would improve significantly and additionally aid in the current development of a vaccine against *Shigella spp*. The high detection rate of these species in samples should be prioritized as vaccination of children in early life could be greatly beneficial [3, 47].

Additionally, although healthcare utilization surveys in the same region in Guatemala demonstrated that a majority of patients sought care for their illness outside the home, less than a quarter of all cases and only 51% of children sought care at a government health facility [23, 48]. This indicates again that our estimates of *Shigella* cases are likely an underestimate of the true burden of disease in the population. Also, though the surveillance platform included the main hospital in each department, the ambulatory facilities were limited in Santa Rosa to five clinics in one municipality (Nueva Santa Rosa) and in Quetzaltenango to three municipalities. Not all patients sought care in those facilities. Patients seen in the emergency department but not admitted to the hospital were not captured by the surveillance system. As a result, our crude and adjusted disease rates may further be an underestimate of the true rates of *Shigella* disease.

## Conclusion

This study demonstrates that *Shigella* is an important cause of enteric infections in Santa Rosa and Quetzaltenango, Guatemala, especially in children < 5 years of age. *Shigella flexneri* and *S. sonnei* were the most common species while *S. dysenteriae* was rare. Antibiotic resistance to commonly used antibiotics was prevalent and complicates treatment. This study demonstrates the importance of strengthening laboratory capacity in Guatemala for determining causes that can lead to prevention of diarrheal diseases, particularly in children. Such capacity building is also critical for rapid detection and control of public health threats at their source, and therefore for global health security. Future steps should include strengthening laboratory surveillance for antibiotic resistance patterns in the region, implementation of common preventive measures, and optimization of oral rehydration therapy.

## Additional files

Additional file 1: Adjusted crude incidence rates of *Shigella* by age and type of healthcare facility in Santa Rosa and Quetzaltenango Departments, Guatemala, 2008–2012. (JPG 206 kb)

Additional file 2: Antimicrobial resistance by site, 2007–2012. (JPG 159 kb)

Additional file 3: Antimicrobial resistance by year from 2007 to 2012. (JPG 197 kb)

## Abbreviations

(MIO) medium: Motility-indole-ornithine
(SIM) medium: Sulfide-indole-motility
ANOVA: Analysis of variance
CDC IEIP: U.S. Centers for Disease Control and Prevention International Emerging Infections Program
CDC: U.S. Centers for Disease Control and Prevention
CLSI: Clinical Laboratory Standards Institute
LIA: Lysine iron
MDR: Multidrug resistance
MSPAS: Guatemalan Ministry of Public Health and Welfare
NARMS: National Antimicrobial Resistance Monitoring System for Enteric Bacteria
PCR: Polymerase chain reaction
SMX/TMP: Trimethoprim/sulfamethoxazole
TSI: Triple Sugar Iron
UVG: Center for Health Studies of the Universidad del Valle de Guatemala
VICo: Vigilancia Integrada Comunitaria
WHO: World Health Organization
XLD: MacConkey and Xylose-lysine-desoxycholate
