# Supplementary figures and images for: Burden of laboratory-confirmed shigellosis infections in Guatemala 2007-2012: results from a population-based surveillance system
Source: BMC Public Health. 2019 May 10;19(Suppl 3):474. doi: 10.1186/s12889-019-6780-7 (PMC6696707; doi:10.1186/s12889-019-6780-7)

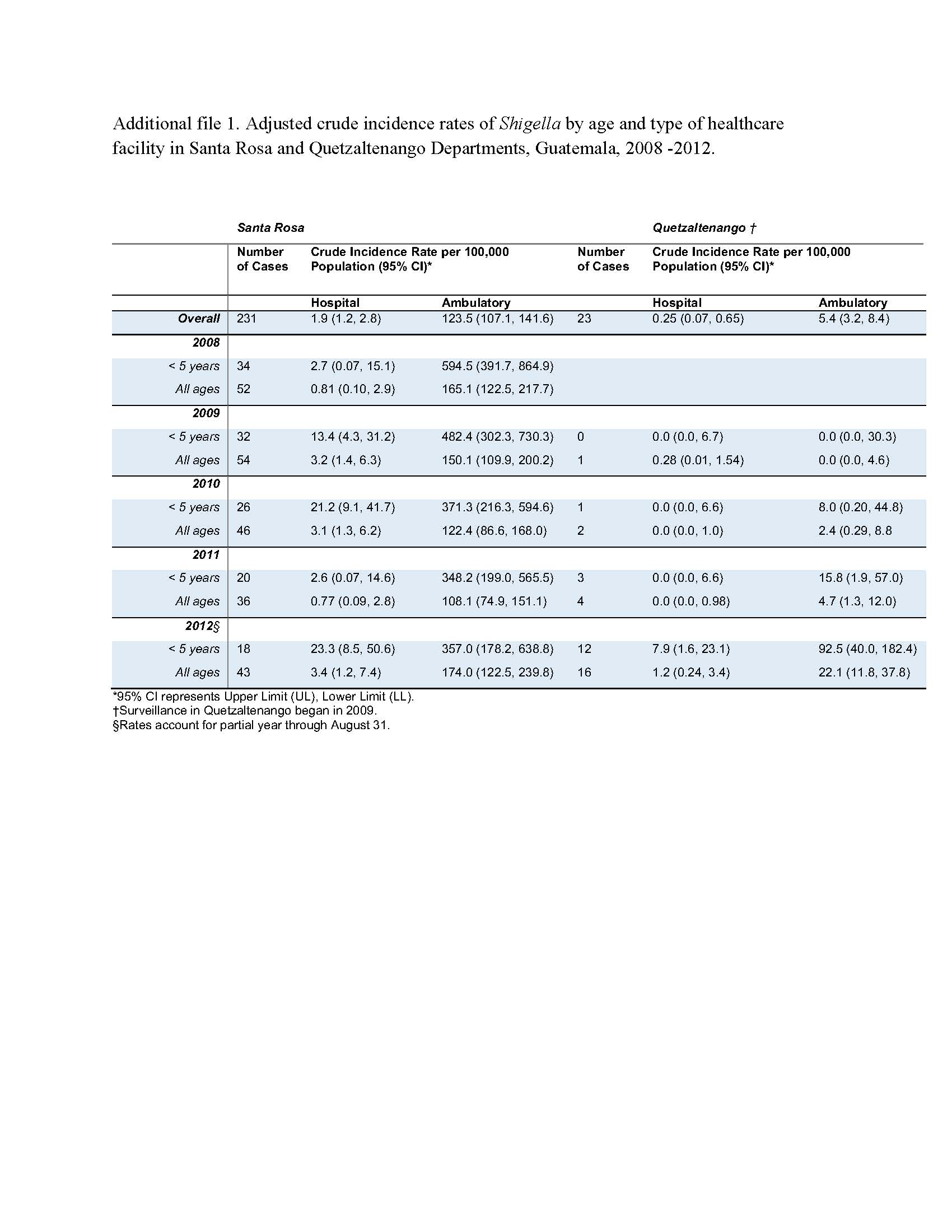

Supplement: Supplementary file 1 — Adjusted crude incidence rates of Shigella by age and type of healthcare facility in Santa Rosa and Quetzaltenango Departments, Guatemala, 2008–2012. (JPG 206 kb) [file 12889_2019_6780_MOESM1_ESM.jpg]

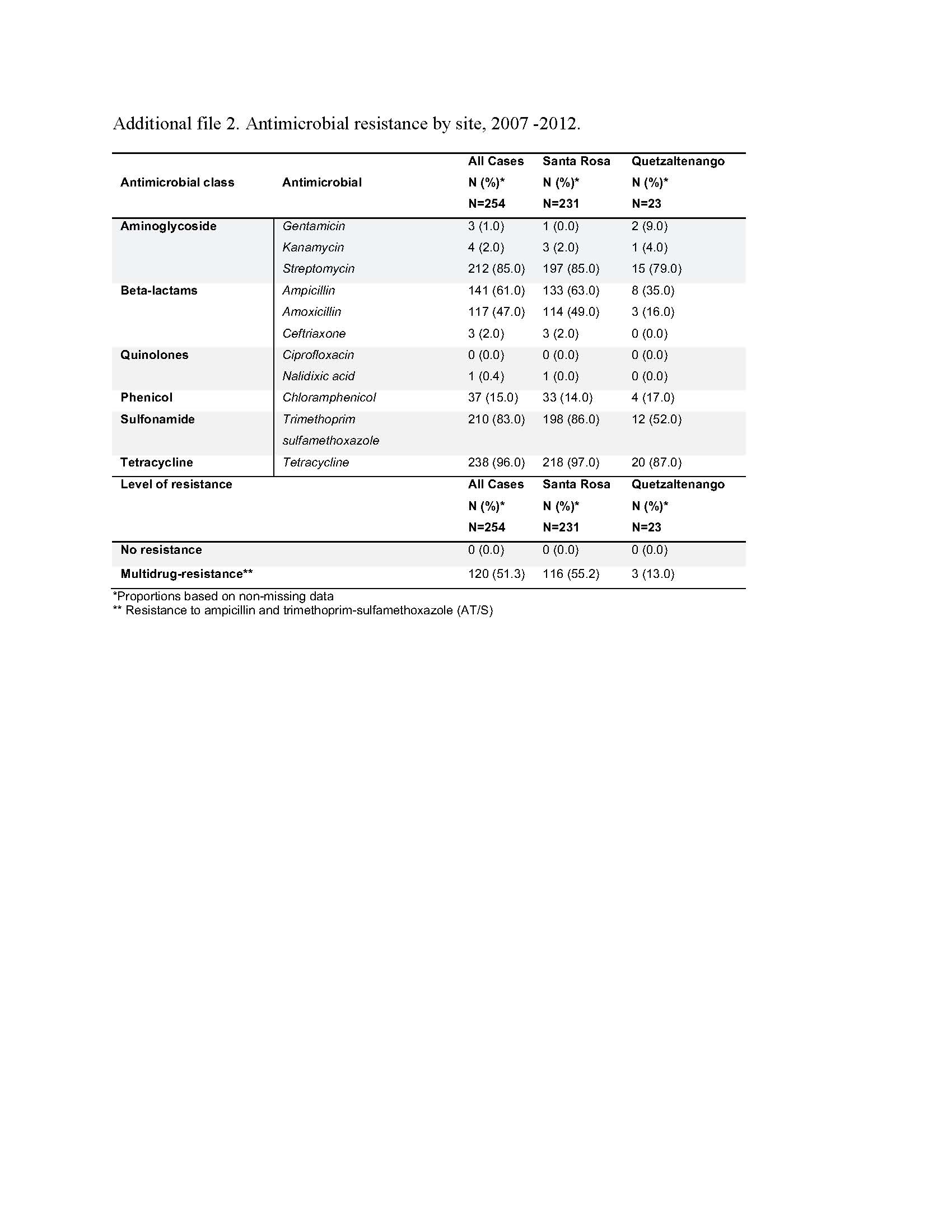

Supplement: Supplementary file 2 — Antimicrobial resistance by site, 2007–2012. (JPG 159 kb) [file 12889_2019_6780_MOESM2_ESM.jpg]

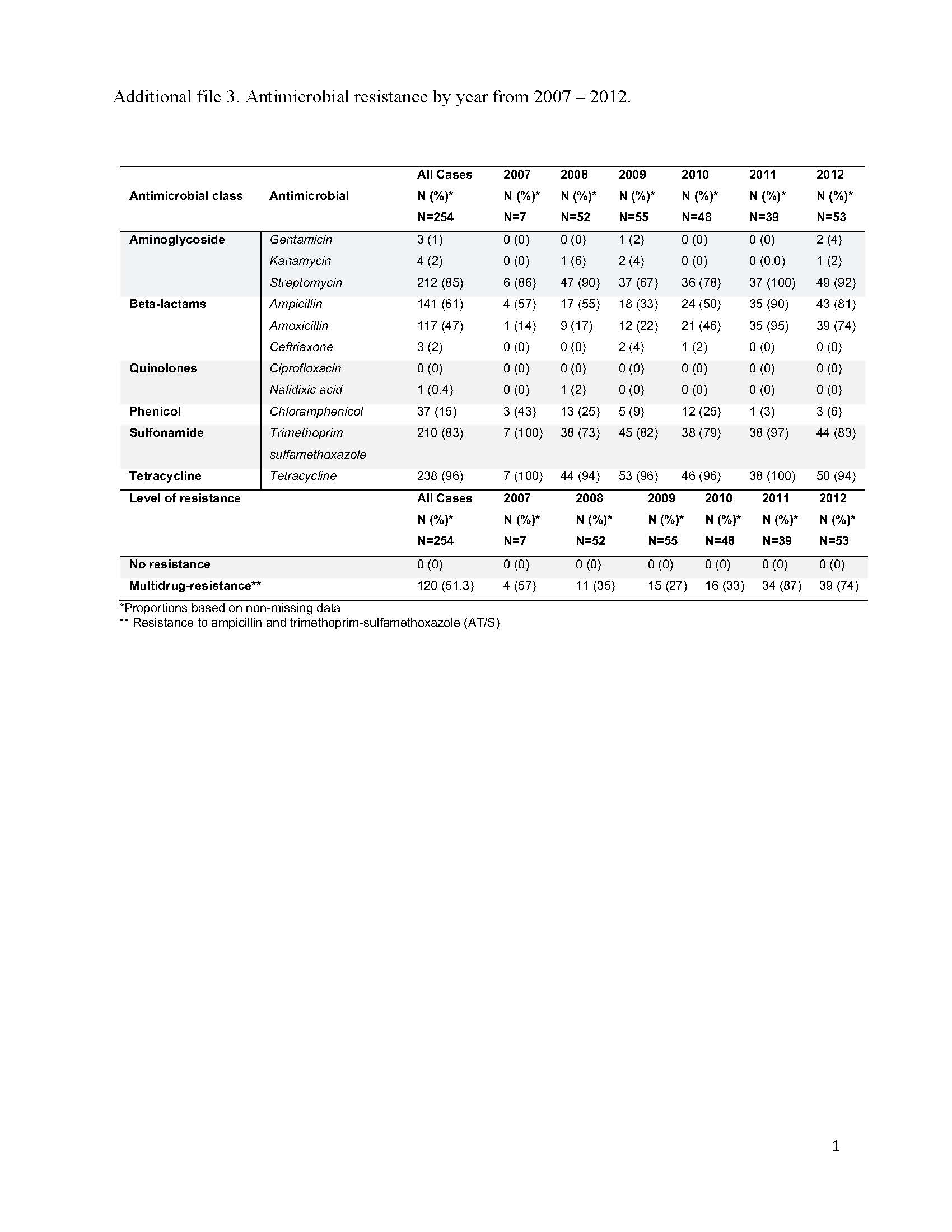

Supplement: Supplementary file 3 — Antimicrobial resistance by year from 2007 to 2012. (JPG 197 kb) [file 12889_2019_6780_MOESM3_ESM.jpg]
